# Supplementary material for: Cholinergic modulation supports dynamic switching of resting state networks through selective DMN suppression
Source: PLoS Comput Biol. 2024 Jun 6;20(6):e1012099. doi: 10.1371/journal.pcbi.1012099 (PMC11185486; doi:10.1371/journal.pcbi.1012099)
Supplement: S1 Text — It includes supplementary figures A,B,C,D,E,F,G,H and supplementary tables A,B,C. (PDF) [file pcbi.1012099.s001.pdf]

Supporting Text Information

Contents

1 SI Figures **2**
1.1 Effects of K+ and AMPA channels modulation in the detailed model. 2
1.2 Effects of K+ and AMPA channels modulation in inhibitory neurons. 3
1.3 Human connectome 4
1.4 Visualization of poorly performing correlation pairs 4
1.5 Selected biophysical variables underlying the BOLD signal and their FC 5
1.6 Pairwise baseline/cholinergic correlation 6
1.7 Changes in human connectome after modulatory ACh change 7
1.8 Effect of cholinergic modulation on FC, auxiliary sortings of the AAL nodes 7

2 SI Tables **8**
2.1 Effect of various transformations on average SC/FC values 8
2.2 AAL parcellation 8
2.3 RSNs definitions in AAL parcellation scheme 9

3 SI References **9**

1 SI Figures

1.1 Effects of K+ and AMPA channels modulation in the detailed model.

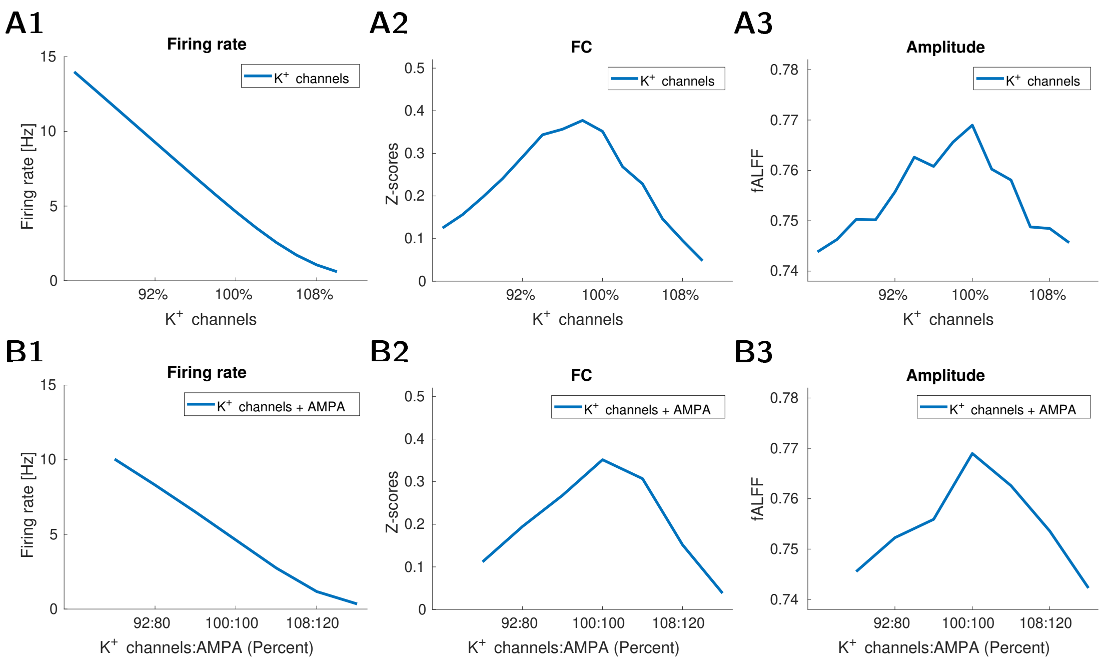

Fig A. Effects of K^+ and AMPA channels modulation in the detailed model. Top. Influence of K^+ channels change (simultaneous change in dendritic potassium leak currents I_K^{leak}, slowly activating potassium M-channel current I_{Km}, Ca^{2+}–sensitive K+ current I_{KCa} in excitatory neurons) on firing rate and FC/fALFF of BOLD. Bottom. Influence of (simultaneous) K^+ channels and AMPA change.

1.2 Effects of K+ and AMPA channels modulation in inhibitory neurons.

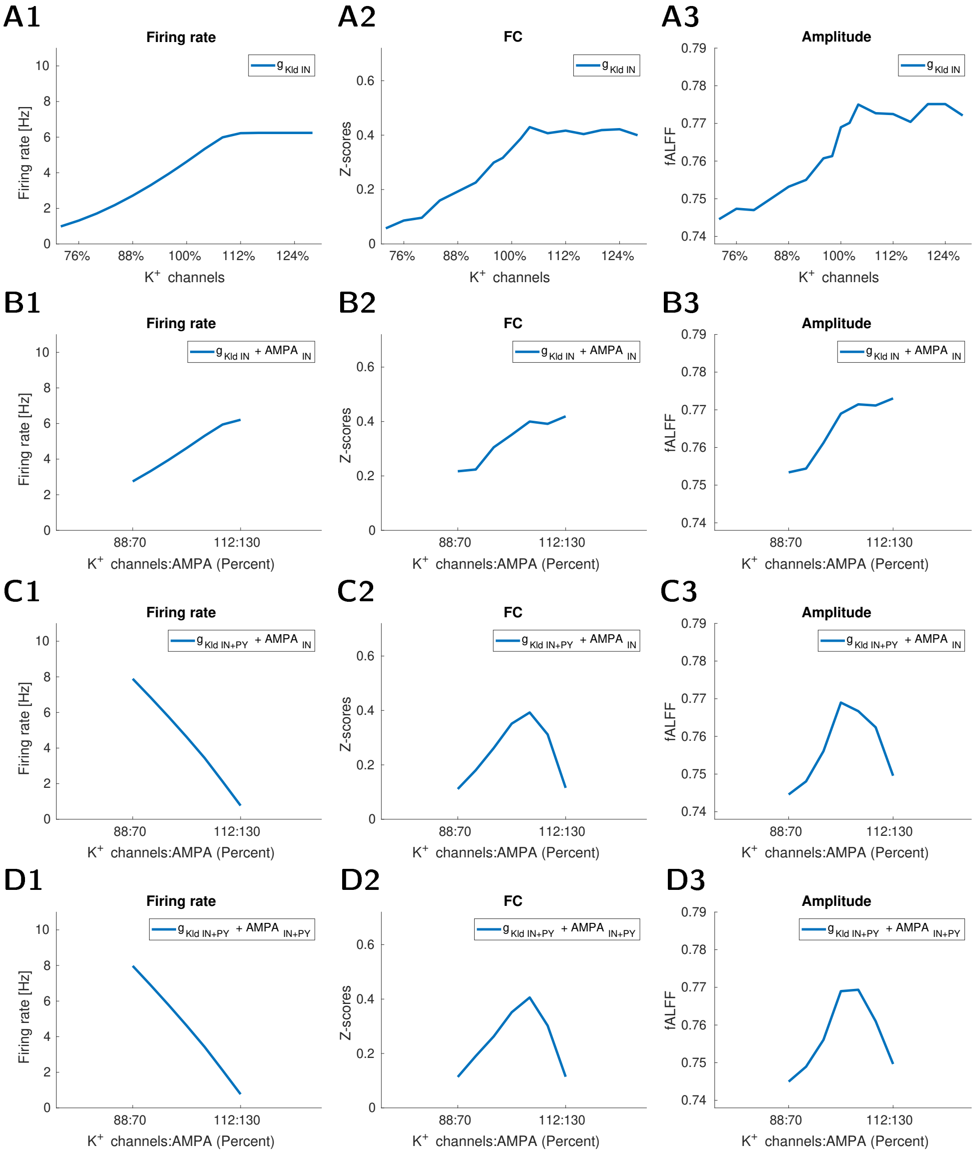

Fig B. Effects of K⁺ and AMPA channels modulation of inhibitory neurons in simplified model. Row A. Modulation of dendritic potassium leak current I_K^{leak} in inhibitory neurons on firing rate and FC/fALFF of BOLD. Row B. Modulation of dendritic potassium leak current I_K^{leak} in inhibitory neurons and simultaneous AMPA change in (excitatory) connections from excitatory to inhibitory neurons. Increase of K⁺ leak changes and AMPA connection to IN cells due to reduction in cholinergic activation on inhibitory neurons resulted in increase of firing rate of excitatory neurons due to overall reduction of inhibition. Row C. Modulation of dendritic potassium leak current I_K^{leak} in inhibitory+excitatory neurons and simultaneous AMPA change in (excitatory) connections between excitatory neurons. Row D. Modulation of dendritic potassium leak current I_K^{leak} in inhibitory+excitatory neurons and simultaneous AMPA change in (excitatory) connections between excitatory neurons and connections (excitatory) from excitatory to inhibitory neurons. Cholinergic modulation applied both to excitatory and inhibitory neurons was qualitatively similar to the only excitatory neurons and did not essentially changed inverted U-shape relationship for FC and amplitude resting-state activity.

1.3 Human connectome

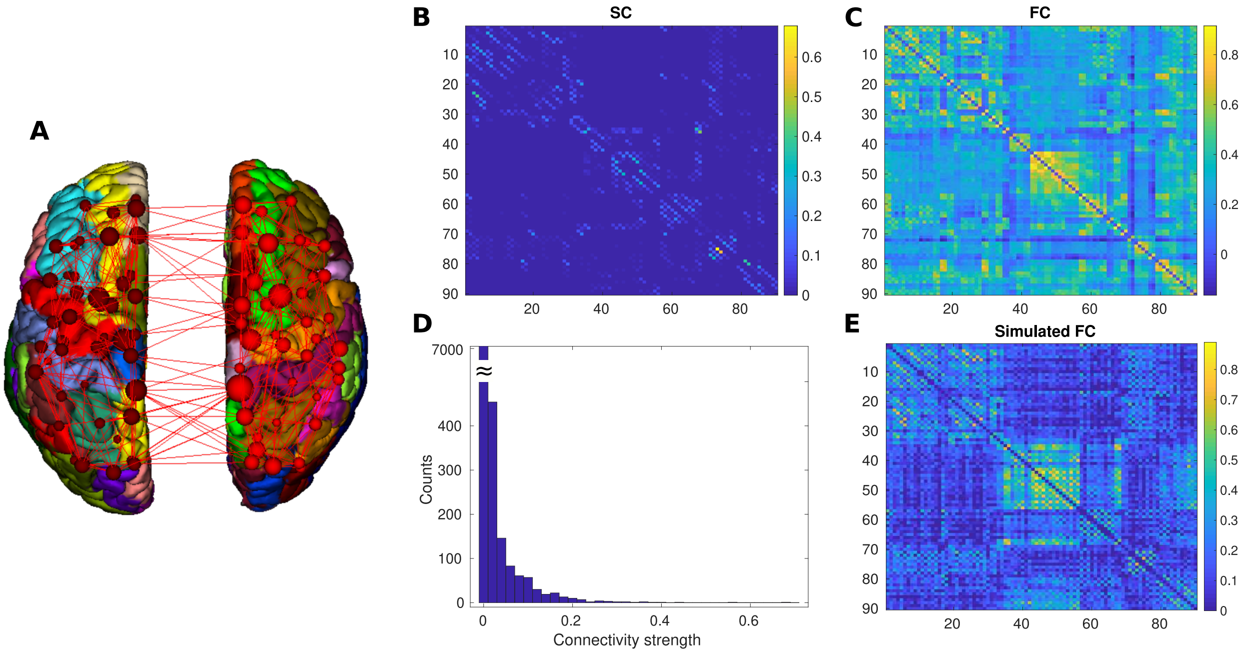

Fig C. Experimental human dataset. A. Visualization of AAL parcelation. B. Average structural connectivity (Both hemispheres, 90 regions, AAL atlas). Large subset of all underlying SCs can be downloaded from [1]. C. Average functional connectivity from identical 90 subjects. D. Distribution of coupling strengths. E. Functional connectivity obtained by simulation of both hemispheres.

1.4 Visualization of poorly performing correlation pairs

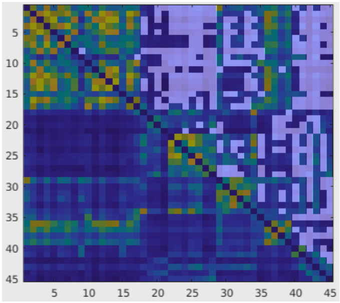

Fig D. Visualization of pair of nodes (white map) for which the model predicts lower correlation than would be expected from experimental fMRI FC. FC from simulated data used as a background.

1.5 Selected biophysical variables underlying the BOLD signal and their FC

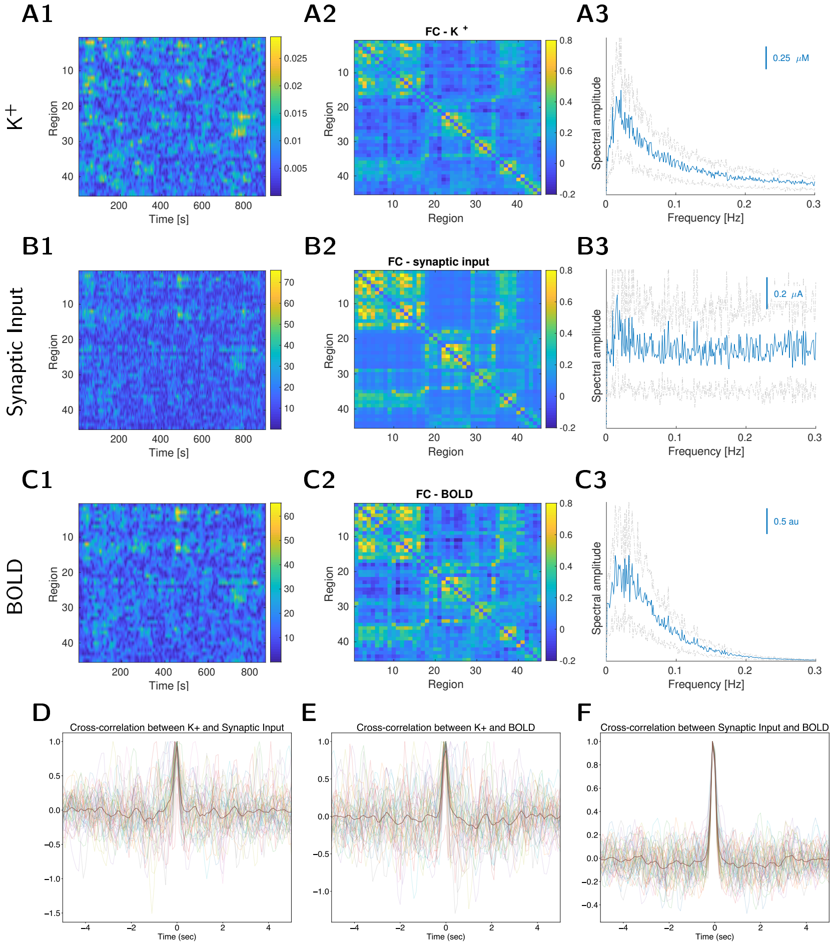

Fig E. Biophysical variables underlying the BOLD. Panels A: extracellular potassium levels (essential for slow fluctuations, see [3]), Panels B: synaptic input (primary source of BOLD). Panels C: resulting BOLD (panels C) dynamics and functional connectivity in the computational model of human connectome. Left panels (1): signal envelope for each region (computed as absolute value of analytical signal obtained from Hilbert transform after the BOLD was filtered between 0.01-0.03 Hz). Middle panels (2) – functional connectivity of raw signal. Right panels (3) – spectral amplitude of signal averaged across all regions (±SD in gray). D/E/F: Crosscorrelation between K+/Synaptic input/BOLD.

1.6 Pairwise baseline/cholinergic correlation

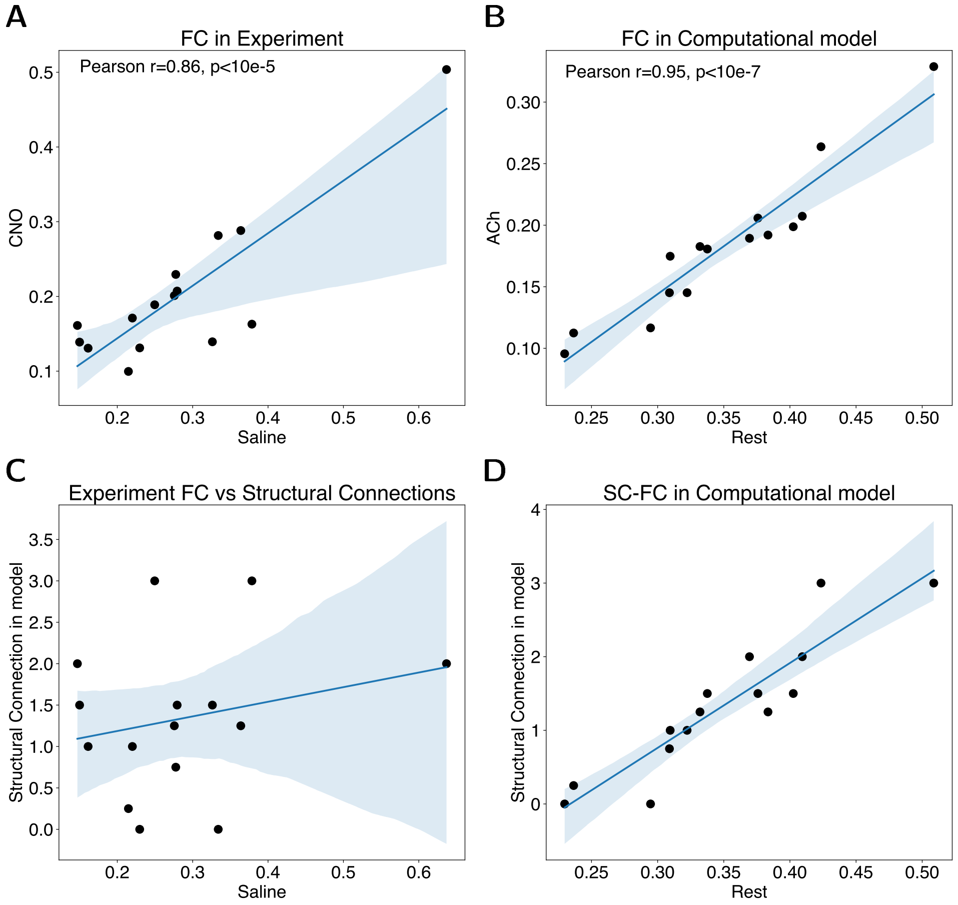

Fig F. Pairwise FC correlation. A. Pairwise comparison of FC values (Fisher z-scores) during saline and CNO condition in experimental recordings. B. Same as A for Rest and ACh conditions in computational model. C. Scatter plot of structural connection strength between pairs of regions used in model with the FC during saline condition in experiment. D. Scatter plot of structural connection strength and the FC during rest condition in the model.

1.7 Changes in human connectome after modulatory ACh change

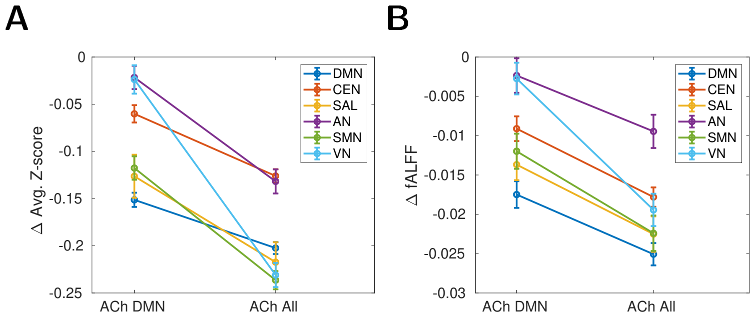

Fig G. Changes in human connectome after modulatory ACh change. The plots are identical with Fig 7 A2/A3, except that we removed from all (non-DMN) networks nodes which are part of DMN.

1.8 Effect of cholinergic modulation on FC, auxiliary sortings of the AAL nodes

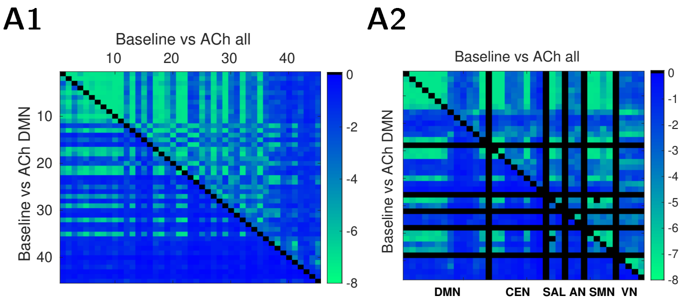

Fig H. Changes in human connectome after modulatory ACh change. FC changes after ACh release in all (upper triangle) / DMN areas (lower triangle). Color meaning as in Fig 7 except of sorting of the areas. A1: Areas sorted by their eigenvector-centrality index. A2: Areas regrouped by their affiliation to different resting-state networks, areas in DMN are omitted in other RSNs, so the influence outside of DMN is better visible.

2 SI Tables

2.1 Effect of various transformations on average SC/FC values

avg of 90 SCs	avg of 90 FCs	Pearson	Spearman	Cosine similarity
90 nodes	90 nodes	0.33	0.27	0.38
symmetric 90	90	0.36	0.27	0.4
45 nodes	45 nodes	0.46	0.38	0.51
symmetric 45	45	0.5	0.39	0.54
symmetric 45, threshold > 0.01	45	0.55	0.57	0.8
symmetric 45, threshold $\leq$0.01	45	0.19	0.21	0.55

Table A. Average correlation of SC/FC matrix for different conditions and transformations. Both hemispheres contain 90 regions. SC is not entirely symmetric, but due to limits of structural imaging the orientation can not be obtained. Thus the symmetric version of connectivity matrix is often considered. The bottom half of the table shows the same comparison for single hemisphere only (45 brain regions). In the last two rows we apply threshold on SC strength, so that either only anatomically "existing" (above threshold 0.01) or "non-existing" (below 0.01) connections are used to compute correlation with their FC counterparts.

2.2 AAL parcellation

Index	ROI name	Index	ROI name	Index	ROI name
1	Precentral	16	Cingulum Ant	31	Parietal Inf
2	Frontal Sup	17	Cingulum Mid	32	SupraMarginal
3	Frontal Sup Orb	18	Cingulum Post	33	Angular
4	Frontal Mid	19	Hippocampus	34	Precuneus
5	Frontal Mid Orb	20	ParaHippocampal	35	Paracentral Lobule
6	Frontal Inf Oper	21	Amygdala	36	Caudate
7	Frontal Inf Tri	22	Calcarine	37	Putamen
8	Frontal Inf Orb	23	Cuneus	38	Pallidum
9	Rolandic Oper	24	Lingual	39	Thalamus
10	Supp Motor Area	25	Occipital Sup	40	Heschl
11	Olfactory	26	Occipital Mid	41	Temporal Sup
12	Frontal Sup Medial	27	Occipital Inf	42	Temporal Pole Sup
13	Frontal Mid Orb	28	Fusiform	43	Temporal Mid
14	Rectus	29	Postcentral	44	Temporal Pole Mid
15	Insula	30	Parietal Sup	45	Temporal Inf

Table B. Mapping between node numbers and their naming in AAL parcellation scheme for a single hemisphere.

2.3 RSNs definitions in AAL parcellation scheme

Functional network	Nodes IDs in AAL parcellation	Covering set in a single hemisphere
DMN (Default mode)	3 4 5 6 7 8 23 24 25 26 31 32 33 34 35 36 37 38 39 40 65 66 67 68 77 78	2 3 4 12 13 16 17 18 19 20 33 34 39
CEN (Central executive)	3 4 7 8 9 14 23 59 61 62 64 65 66 67 72 77 85 89	2 4 5 7 12 30 31 32 33 34 36 39 43 45
SAL (Saliency)	7 8 19 20 29 30 31 32	4 10 15 16
AN (Auditory)	78 79 80 81 82	39 40 41
SMN (Sensimotor)	1 2 20 57 58 69 77 78	1 10 29 35 39
VN (Visual)	43 44 49 50 51 52 53 54 77	22 25 26 27 39

Table C. Definitions of functional networks in the AAL template taken from [2]. Direct mapping on AAL ids is courtesy of Won Hee Lee and Sophia Frangou. The most-right column is the reduction on a single hemisphere (45 nodes, in case of the few non-symmetric nodes we use the covering set).

3 SI References

1. Škoch A, Rehák Bučková B, Mareš J, Tintěra J, Sanda P, Jajcay L, et al. Human brain structural connectivity matrices–ready for modelling. Scientific Data. 2022;9(1):1–9.

2. Lee WH, Frangou S. Linking functional connectivity and dynamic properties of resting-state networks. Scientific Reports. 2017;7(1):1–10.

3. Krishnan GP, González OC, Bazhenov M. Origin of slow spontaneous resting-state neuronal fluctuations in brain networks. Proceedings of the National Academy of Sciences. 2018;115(26):6858–6863.
