# Supplementary material for: Cholinergic modulation supports dynamic switching of resting state networks through selective DMN suppression
Source: PLoS Comput Biol. 2024 Jun 6;20(6):e1012099. doi: 10.1371/journal.pcbi.1012099 (PMC11185486; doi:10.1371/journal.pcbi.1012099)
Supplement: Attachment — Submitted filename: pcb_response.docx [file pcbi.1012099.s002.docx]

**Response to Reviewers**

We would like to thank the reviewers for their recognition of the strengths of the paper and very helpful critiques. In response to the reviewers’ comments we have addressed the comments (marked by violet font) with changes to the manuscript (marked by red font) based on additional simulations and analyzes.

**Reviewer #1:**

The authors investigate the neural mechanisms underlying the modulation of resting-state brain activity by cholinergic neuromodulation. Using a computational model, the authors demonstrate that changes in excitability and recurrent connections influenced by cholinergic modulation impact resting-state activity. The results align with experimental findings in rodents regarding cholinergic modulation of the Default Mode Network (DMN). The study also extends its scope to human resting-state simulations, revealing that increased cholinergic input leads to a reduction in functional connectivity across the brain. Moreover, selective cholinergic modulation of the DMN mirrors observed transitions between baseline resting state and states with suppressed DMN fluctuations associated with attention to external tasks.

This model is useful tool to explore the functioning of neuromodulation mechanisms and sub-networks activations in the brain. For example, the authors conducted interesting experiments on neuromodulation of specific sub-networks and investigated the role of modulating specific parameters (e.g. K+ leak conductance and PY-PY AMPA conductance).

This research provides valuable insights into the neural mechanisms underlying the effects of cholinergic neuromodulation on resting-state brain activity and its dynamics, and on transitions between states with externally and internally oriented attention, which are still poorly understood.

Given the computational nature of this study, it's crucial for the authors to achieve a reliable validation of the results.

My major concern is that some observations on the model might not be easily confirmed through experiments, and it might not be easy to confirm their significance.

The tested hypothesis on neuromodulation could, in principle, work by chance, and not be the correct mechanism happening in the brain. Also, it could be distorted by the difficulty to constrain the model (e.g. given the experimental limitations in recording human connectome).

**Major:**

-line 77: “*We used experimental results of direct cholinergic neuron manipulation to constrain our model of the DMLN network.*” However, it is not clear to me exactly how the model is constrained. Maybe the authors could discuss this in more detail? To have a reliable tool to constrain models on experimental data is an open issue, and to demonstrate this could be a strength point of the work.

The way the model was constrained could indeed be better described in the manuscript. As the reviewer correctly pointed out, constraining models is challenging and an open problem, which is further made worse with the large number of parameters in realistic models. While we do not have an ideal solution to this problem, in this work we used selected parameter sweeps that are informed from experiment and theories to constrain model parameters to match experimental observations. Below we provide the motivation and some results of various parameter sweeps, which we now include in the manuscript.

Our primary variables of interest in experiments and computational model is the functional connectivity and amplitude of low-frequency fluctuations (in resting-state frequencies). Thus, we performed various informed parameter sweeps to examine how model parameters impacted the above measures. In addition, we also monitored that the Na^+^, K^+^ ion concentrations, Na^+^/K^+^ pump and synaptic input stayed within their permitted range (ion concentrations stayed within observed values from past experiments) and there were no pathological (e.g. seizure-like) activity in network dynamics.

In order to reduce the number of parameters to examine, we made informed choice on the parameters to examine. In particular, based on our previous work (Krishnan et al., 2018), we examined parameters that influences the extracellular ion dynamics and synaptic connections which is known to have large impact on resting-state activity. At the same time we also know from past cellular level experiments (eg. work of McCormick et al.) that cholinergic modulation affects potassium currents (detailed account of these is provided at section 2.1 and in the next response) and excitatory synaptic connections. Thus, we examined the conductances of K^+^ currents (including K^+^ leak, Km, Ca^2+^-sensitive K^+^ currents) in excitatory and inhibitory neurons that could be influenced by cholinergic modulation and have an impact on the ion dynamics. Similarly, the we also varied excitatory and inhibitory synaptic connections to examine its impact on resting-state activity which are influenced by cholinergic modulation.

We then performed several (1-D or 2-D) sweeps which revealed the qualitative nature and range of parameter values that best matched the results from experiments. The simple sweeps (see new figure below) showed that modulation of all K^+^ currents modulation resulted in an inverted U-shape dependency for both functional connectivity and amplitude. A similar exploration was performed with inhibitory connections (see SI Fig. 12 and response to reviewer 2). Combining the modulation of all K^+^ current and AMPA conductance resulted in similar qualitative pattern, with the inverted U-curve slightly shifted (the same was later observed with simplified model in 2-D sweep of Fig. 9). The parameters corresponding to firing rate around 5Hz matched the peak of the inverted U-curve in the resting-state activity amplitude, which were taken as the 100% or rest condition. As the cholinergic modulation is known to reduce K^+^/AMPA channel conductance, the parameters corresponding to the left of the peak and that was close to experimental FC/amplitude were taken as cholinergic condition. Admittedly this procedure does not examine the whole space of parameter values, but the qualitative patterns observed were robust across variations of other parameters, consistent with cellular electrophysiological studies on cholinergic modulation and provide a hypothesis that can be tested in future experiments.

We made the following change to the main text:

By examining the impact of change in each of the ionic and synaptic currents on resting-state activity, we identified that K^+^ leak and AMPA current had the largest impact on resting-state activity, see Fig. 3 (detailed treatment of K^+^ currents is in SI Fig. 11 and effects of modulating inhibitory population is in SI Fig. 12).

We also included the following description and new figure in the method section of the manuscript and new figure into supplementary material:

We performed various (1-D or 2-D) sweeps which revealed the qualitative nature and range of parameter values that best matched the results from experiments. The simple sweeps (see SI Fig. 11) showed that modulation of all K^+^ currents modulation resulted in an inverted U-shape dependency for both functional connectivity and amplitude. A similar exploration was performed with inhibitory connections (see SI Fig. 12). Combining modulation of all K^+^ current and AMPA conductance resulted in similar qualitative pattern, with the inverted U-curve slightly shifted (the same was later observed with simplified model in 2-D sweep of Fig. 9). The parameters corresponding to firing rate around 5Hz matched the peak of the inverted U-curve in the resting-state activity amplitude, which were taken as the 100% or rest condition. As the cholinergic modulation is known to reduce K^+^/AMPA channel conductance, the parameters that were left of the peak and close to experimental FC/amplitude were taken as cholinergic condition. Admittedly this procedure does not examine the whole space of parameter values, but the qualitative patterns observed were robust across variations of other parameters, consistent with cellular electrophysiological studies on cholinergic modulation and provide a hypothesis that can be tested in future experiments.

Figure 11: Effects of K+ and AMPA channels modulation in the detailed model. Top. Influence of K+ channels change (simultaneous change in dendritic potassium leak currents I^leak^_K_, slowly activating potassium M-channel current I_Km_, Ca2+ –sensitive K+ current I_KCa_ in excitatory neurons) on firing rate and FC/fALFF of BOLD. Bottom. Influence of (simultaneous) K+ channels and AMPA change for (excitatory) connections between excitatory neurons.

- I have a general concern how what is the qualitative evaluation of the model. E.g. to justify the validation of the model, the authors write: *We then used a computational framework that sufficiently mimicked the experiments* (line 127). But it is not clear to me if there is a quantitative evaluation of this comparison. They refer to Fig 9 in SI, but this is an important point that should be better discussed in the main text.

We thank the reviewer for pointing the lack of clarity in our description. By “*computational framework*” we meant the constraining the model parameters to qualitatively match the model’s averaged functional connectivity and amplitude of low-frequency fluctuations (measured by fALLF) with the experiment across different conditions.

We also note that by “computational framework” we did not mean any specific tool to constrain models on experimental data. Since, we used a biophysically realistic model, optimization techniques are compute intensive and infeasible in some cases (to give an idea - our largest investigation of this model was (just) 2-D sweep of human connectome to check E/I stability similar to current Fig. 9. Even such simple sweep takes about 3 weeks of parallel computations on computer cluster with 800 CPU cores). Given the computational limitations and stability of model behavior in investigated ranges we were able to qualitatively match the model behavior with experimental data (e.g. Fig. 4.). Our further exploration suggests that optimization could improve match, but the qualitative results and predictions in our study will not change. In the revised manuscript, we rephrased these sentences for better clarity.

We agree with the reviewer on the importance of the result in Fig 9 which was previously included in the supplement. In this figure, we examined the different possible effects of cholinergic modulation, by comparing a detailed and a reduced model. Following the suggestion of the reviewer, we moved the figure to the main text and changed the structure of the manuscript to emphasize this evaluation.

The following are the changes in the manuscript:

1) We moved the paragraph describing the detailed model of cholinergic modulation to section 2.1, and joined it with the text describing former SI Fig. 9, now moved from SI to the main text as a new Fig. 3.
2) “We then used a computational framework that sufficiently mimicked the experiments” was replaced by “The computational simulations modeling the experiments were setup in the following way.”

The relevant section now reads as follows:

“The computational simulations modeling the experiments were setup in the following way. The connectivity of rat DMLN was used and synaptic activity from each anatomical area was used to derive BOLD signals, see Fig. 2B. We compared the simulation during baseline resting state and during high ACh levels that correspond to the experimental post-saline baseline and CNO conditions, respectively (Fig. 2B). We monitored several dynamic variables of the model – including firing rate, the activity of Na^+^/K^+^ pump, extracellular K^+^ levels, neuronal membrane voltage (spiking) and synaptic input. The total synaptic input was transformed into a BOLD signal representing the dynamics of each brain area (see Methods).

Past experimental work has shown that acetylcholine modulates several ionic currents and synaptic currents through nicotinic and muscarinic receptors. Based on these findings, we implemented a detailed model of cholinergic modulation as a reduction of somatic and dendritic potassium leak currents IK leak , somatic delayed- rectifier potassium current IKv , slowly activating potassium M-channel current Ikm , high-threshold Ca2^+^ current IHVA , Ca^2+^ –sensitive K^+^ current IKCa in excitatory neurons based on past experimental work [McCormick and Prince, 1986, McCormick, 1992, McCormick et al., 1993, Galvin et al., 2020]. In inhibitory neurons, the somatic and dendritic IK leak current and somatic Ikv current were reduced. Further, the influence of ACh on synaptic transmission was implemented as a decrease in excitatory AMPA connections and increase in NMDA connectivity based on experimental work [Gil et al., 1999, Hsieh et al., 2000, Vijayraghavan et al., 2018]. By examining the impact of change in each of the ionic and synaptic currents on resting-state activity, we identified that K^+^ leak and AMPA current had the largest impact on resting-state activity, see Fig. 3 (detailed treatment of K+ currents is in SI Fig. 11). Adding cholinergic modulation to inhibitory neurons did not have large impact in our model (effects of modulating inhibitory population is shown in SI Fig.12). This may be partly due to use of a canonical type of inhibitory neuron and future studies are required to implement different subtypes of inhibitory neurons (e.g. Lu et al., 2017). All these findings allowed us to use a simplified model with changes to only K^+^ leak and AMPA currents in excitatory neurons and made possible the analysis of cellular mechanisms (detailed in Fig. 9). We use this simplified model in all subsequent sections.

After performing systematic variations of K^+^ leak and AMPA conductance we identified that a reduction of 8% in K^+^ leak currents and a 20% reduction in AMPA currents (local and long-range connections) qualitatively matched the results observed in DREADD experiments (i.e. changes in functional connectivity and spectral amplitude).“

Regions decorrelate, after achetilcoline injection (simulation of increased excitability). But do they decorrelate in a similar way? Are there regions that decorrelate more than others? To be constructive, I would suggest measuring the correlation between the change in FC in model and simulations.

We thank reviewer for the question and the constructive suggestion. Following the reviewer’s proposal, we performed few different comparisons between the changes in FC in the experiment and the model. First, we examined the correlation between the FC pairs of regions during saline condition in experiment (or rest in model) compared to CNO condition (ACh condition in model). In both cases, we observed a strong positive correlation between the two conditions. This suggests that there is a large influence of the baseline FC in both experiment and the model. The regions which are strongly coupled in saline (or rest) remained strongly coupled following CNO (or ACh), but with lower strength. This correspondence demonstrate the same internal consistency in the qualitative nature of the change in FC following cholinergic modulation in model and experiment. Second, we examined how well structural connections (SC) used in the model captured the FC during saline in experiment and rest condition in model. While there was a strong correlation between the SC and FC in the model, it was not significant in comparison to the saline condition in the data. Thus, this suggest that the SC we used in the model may lack additional details of the DMLN network (notably there is no full rat’s connectome publicly available and thus interaction with the rest of the network is missing). Consequently the direct comparison of the pairwise FC between experiment and the model did not yield significant results (r~0.2, p>0.05). This effect is largely due to the discrepancy between experimentally measured structural connection and the FC for DMLN in rodents. If we had used the saline FC as our structural connection in the computational model, then we will have strong correlation between the pairwise comparison of regional FC between model and experiment as reflected by the strong correlation between rest condition and SC in the model.

Based on these new results, we included the following text to the result section and a supplementary figure:

“We examined the correlation between the FC pairs of regions during saline condition in experiment (or rest in model) compared to CNO condition (ACh condition in model) (Fig. SI 16 A, B). In both cases, we observed a strong positive correlation between the two conditions. This suggests that there is a large influence of the baseline FC in both experiment and the model. The regions which are strongly coupled in saline (or rest) remained strongly coupled following CNO (or ACh), but with lower strength. This correspondence demonstrate the same internal consistency in the qualitative nature of the change in FC following cholinergic modulation in model and experiment. However, due to low correlation between structural connection used in the model and FC during saline condition (Fig. SI 16C), we did not observe significant correlation between model and experiment for the pairwise FC measure. This suggest that the SC we used in the model may lack additional details of the DMLN network - notably there is no full rat’s connectome publicly available and thus interaction with the rest of the network is missing.”

We added new figure 16 in section 5.9 of supplementary material:

Figure 16: Pairwise FC correlation. A. Pairwise comparison of FC values (Fisher z-scores) during saline and CNO condition in experimental recordings. B. Same as A for Rest and ACh conditions in computational model. C. Scatter plot of structural connection strength between pairs of regions used in model with the FC during saline condition in experiment. D. Scatter plot of structural connection strength and the FC during rest condition in the model.

- In fig5 the change in FC is shown in the model, but there is no comparison with the data. This is due to the lack of human data for that condition. However, is it possible to validate the result in some way? For example, the increase of correlation between FC and SC, is it observed also experimentally in some condition?

Indeed, a direct comparison with specific experimental result would be helpful to validate this result. However, it is worth noting that the goal of computational modeling is go beyond the experimental results to identify potential hypothesis which can be examined in future work. And in the case of SC/FC change with cholinergic modulation (also in the eigenvector centrality measure case), the computational model provides an intriguing but a consistent prediction for which we were not able to find direct experimental evidence. Below we provide some related experimental work, but given the evidence is indirect, we include only a brief discussion about this in the manuscript. We hope that future experimental work is motivated through our model, which can be proved or disprove the thesis in this work.

We found three indirect cases of evidence that relate to our observations. First, several studies examining the activity in resting-state frequencies have observed changes in the functional correlations during task (eg. Damoiseaux et al., 2006). While the change in correlation between FC and SC is not reported, assuming that SC remains constant during task and rest period, and task often involves cholinergic changes (Everitt et al., 1997), it is suggestive that FC-SC also changes from rest to task (potential change in cholinergic modulation). Second, Straathof et al., 2019 presents systematic review of the literature examining SC-FC correlation and observed this correlation varied anywhere between 0.2-0.8 for group level analysis (Fig. 2 in there). This suggests a large methodological and inter-individual variability in this measure. One possibility is that the variability arises from difference in subject’s state and the experimental condition that can influence subject states thus supporting the SC-FC change with different internal states. Finally, the SC-FC coupling is found to be reduced in Alzheimer disease (AD) (Sun et al., 2014). Since, cholinergic neurons in BF are severely lost during AD progression (Ferreira-Vieira et al., 2016) and reduction of cholinergic signaling is suggested to play significant role in AD (Chen at al., 2022), this is indirect evidence suggesting that cholinergic activation increases SC-FC correlation.

We added the following brief discussion to the manuscript:

Few cases of indirect evidence suggest that SC-FC could be influenced by cholinergic modulation. It is known that FC changes during task compared to resting-state (Damoiseaux et al) and that task-related activity is often accompanied by change in cholinergic modulation (Everitt et al., 1997). Assuming structural connections remains the same, it is likely the SC-FC also changes from resting-state to task. Further, some of the variability of SC-FC observed across subjects and studies (Straathof et al., 2019) may be due to the state of subject and provide support to our observation. Finally, the reduction of SC-FC in Alzheimer’s disease (Sun et al., 2014), where there is a reduction of cholinergic activation (Ferreira-Vieira et al., 2016; Chen at al., 2022) provide additional support to our findings.

- Same for the change as a function of eigenvector centrality. Can this be compared with any experimental finding?

Similar to the previous response, we were not aware of direct experimental evidence for results on eigenvector centrality. We are aware of attempts to relate eigenvector centrality on connectivity data in Alzheimer disease (Lorenzini et al., 2023), but only on functional connectivity (as is usual, e.g. [Zuo et al., 2012](doi:10.1093/cercor/bhr269)), so we can’t directly relate to our results. It seems well possible that the structural data are privately available for similar datasets and (in)validation of our prediction is in principle possible to be verified. We thus emphasized in the manuscript possibility of testing our predictions on existing datasets.

We included the following to the manuscript:

Relating SC-FC changes as a function of eigen-vector centrality is not common place in the literature but it might be worth to test our predictions on possibly related datasets (e.g. Lorenzini et al., 2023).

-line 231: “*Thus a selective cholinergic modulation of DMN would support the switch between externally oriented task states to internally oriented states*”. For my understanding, the authors are saying that DMN only modulation does not affect sensory networks, but I don’t understand how this can demonstrate the link to the internally oriented states. Could the authors discuss better this point?

We should have provided better explanation about this in the manuscript and we thank the reviewer for this question. The link to internally/externally oriented states is based on several experimental observations suggesting that DMN is a) involved in internal forms of mentation and b) antagonistic to other networks engaged during externally oriented tasks (Buckner et al., 2019). We also identified a recent paper Harrison et al. 2022 that provided direct evidence for selective increase in DMN activity during internal oriented states / rest (participants in internal, self focused, task) and DMN suppression during external oriented states (participants transitioned from the rest to externally focused task). In addition, Harrison et al. results also provide experimental evidence of correlation between BF activity (source of ACh) and DMN.

This is consistent with our hypothesis based on computational model. If DMN activity is a marker of internal mentation and selective ACh release inhibits this network, it is favorable that the release does not inhibits sensory networks at the same time, so that antagonistic networks dealing with external-sensory environment can be independent/active. In the opposite case we would conclude that selective targeting of DMN would inhibit both internally/externally engaged networks.

The changes in the manuscript:

We rewrote the description on line 231 and include the reference to the discussion:

These findings demonstrate that selective cholinergic modulation of DMN has brain-wide changes in resting-state activity, with the notable exception of the sensory networks. If DMN activity is a marker of internal mentation and selective ACh release suppresses only DMN, it is favorable that the release does not suppress the sensory networks at the same time, so that antagonistic networks dealing with external-sensory environment can be decoupled/active. Thus, we hypothesize that selective cholinergic modulation could be one of the neural processes that facilitates the transition from internal to external oriented states (see Discussion for related experimental results).

We also include the relevant discussion in the manuscript :

 “In human connectome simulations, we concentrated on a particular role of ACh in switching the balance in the neocortical state from default mode network activation to activation of networks involved in external sensory processing [Tanabe et al., 2011, Klaassens et al., 2017] or executive control [Sutherland et al., 2015]. DMN, a network active mainly in the resting condition [Greicius et al., 2003], was associated with a variety of internally oriented mental states [Andrews-Hanna et al., 2014, Konishi et al., 2015] while inhibited with external goal-oriented tasks [Fox et al., 2005, 2009, Andrews-Hanna et al., 2014]. A recent experimental study (Harrison et al., 2022) observed DMN suppression when participants transitioned from the rest to externally focused task and DMN activation during internal, self focused, task. At the same time there was a correlation between DMN and BF activity (source of ACh), suggesting a potential role of cholinergic modulation. In our simulations we considered global cholinergic modulation of the entire brain and selective cholinergic modulation only in DMN. Cholinergic modulation only in DMN regions translated to a picture consistent with the experimental results in rodents [Peeters et al., 2020]; moreover, it showed DMN-speciﬁc inhibition which did not appear when ACh was uniformly aﬀecting all cortical regions. Within the DMN, the modulation mainly aﬀected the areas with higher eigenvector-centrality rank (i.e., highly connected nodes preferring connections to other highly connected nodes). As DMN richly connects (and even shares some nodes) with other functional networks, ACh-triggered changes in DMN propagate to other parts of the brain, however to a lesser degree than it is the case of uniform cholinergic release across all the areas; notably, the change did not impact internal coupling within visual/auditory networks. This observation makes cholinergic-related suppression of DMN compatible with independent activity in sensory regions connected with attention to external stimuli.”

**Minor:**

- If I am not wrong, CNO is not introduced when firstly mentioned.

You are correct, we first use it at line 66, where we say “In particular, the injection of a synthetic drug (CNO) designed to activate DREADD receptors expressed on BF cholinergic neurons”...

We changed that to:

“In particular, the injection of a synthetic drug (clozapine-N-oxide, CNO) designed to activate DREADD receptors expressed on BF cholinergic neurons”...

- Fig3, it could be made explicit in the figure that the two lines refer to data and model (maybe it could be explicitly written on the left of the panels, or using a thumbnail).

“Data” and “Model” labels were added to the left side of the panels of Fig 4 (formerly Fig. 3).

- In fig5 caption it might be more explicit that this is a results on simulation.

Fig 6 (formerly Fig. 5) caption started with: “Effect of generic cholinergic release on the connectivity (each condition 20 trials).”

It was changed to: “Effect of generic cholinergic release on the connectivity (each condition 20 simulated trials).”

**Reviewer #2:**

The authors have tried to delineate the role of cholinergic neuromodulation on brain activity during resting state by a combination of integrating experimental data and simulations in rodents and human subjects. The mechanisms of why low-frequency fluctuations in brain activity are greater particularly during resting periods as opposed to active periods are investigated.

A dominant resting state sub-network called the default-mode network (DMN) is described, which shows an increase in functional connectivity upon a reduction in cholinergic release, which also results in an increase in resting state brain activity. The authors further demonstrate using human resting-state simulations that brain-wide functional connectivity is reduced with increase in cholinergic input.

The study utilizes a biophysical network model of the rat and human connectome. In particular, the authors crucially demonstrate using computational modelling that changes in cellular excitability (cellular intrinsic properties) and recurrent connectivity (synaptic changes) by cholinergic modulation results in greater low-frequency fluctuations in brain activity during resting periods.

Some points that need more clarity:

1. The model has excluded the influence of cholinergic modulation on different cell types and focused primarily on excitatory connections. Is this oversimplified and leading to a biased result in the modeling?

We thank reviewer for this important comment. It is known that ACh affects the inhibitory neurons as well (e.g. Kawaguchi, 1997). Although we did not originally present the simulations involving inhibitory neurons in the manuscript we had performed several simulation examining the effect on inhibitory neurons and per reviewer suggestion we will now include new Fig. SI 12 showing this effect. In our model, an increase of K+ leak changes and AMPA connection to IN cells due to reduction cholinergic modulation on inhibitory neurons resulted in increase of firing rate of excitatory neurons due to overall reduction of inhibition. Further, the cholinergic modulation applied both to excitatory and inhibitory neurons was qualitatively similar to the only excitatory neurons and did not essentially changed inverted U-shape relationship we observe when modulating only the excitatory part of the circuit (see figure below, bottom row). Thus, for our model we mainly focused on the excitatory neurons. However, we do note that our model lack inhibitory neuron sub-types that could have larger influence of network activity, eg. chandelier neurons that innervate the axon initial segment and receives cholinergic input (Lu et al., 2017). Thus, future work is required to examine the role of inhibition in more systematic way.

We modified the following text in the manuscript:

Adding cholinergic modulation to inhibitory neurons did not have large impact in our model (effects of modulating inhibitory population is shown in SI Fig.  12). This may be partly due to use of a canonical type of inhibitory neuron and future studies are required to implement different subtypes of inhibitory neurons (e.g. Kawaguchi, 1997; Lu et al., 2017).

We added the following figure in the supplementary material:

#### Effects of K+ and AMPA channels modulation in inhibitory neurons.

Figure 12: Effects of K^+^ and AMPA channels modulation of inhibitory neurons in simplified model. Row A. Modulation of dendritic potassium leak current I_Kleak_ in inhibitory neurons on firing rate and FC/fALFF of BOLD. Row B. Modulation of dendritic potassium leak current I_Kleak_ in inhibitory neurons and simultaneous AMPA change in (excitatory) connections from excitatory to inhibitory neurons. Increase of K^+^ leak changes and AMPA connection to IN cells due to reduction in cholinergic activation on inhibitory neurons resulted in increase of firing rate of excitatory neurons due to overall reduction of inhibition. Row C. Modulation of dendritic potassium leak current I_Kleak_ in inhibitory+excitatory neurons and simultaneous AMPA change in (excitatory) connections between excitatory neurons. Row D.  Modulation of dendritic potassium leak current I_Kleak_ in inhibitory+excitatory neurons and simultaneous AMPA change in (excitatory) connections between excitatory neurons and connections (excitatory) from excitatory to inhibitory neurons. Cholinergic modulation applied both to excitatory and inhibitory neurons was qualitatively similar to the only excitatory neurons and did not essentially changed inverted U-shape relationship for FC and amplitude resting-state activity.

2. The authors use resting state fMRI data as a measure of neuronal activity. It would be good to describe how fMRI data actually correlates to this, in comparison with cellular-level and large-scale neuronal population-level activity by other methods?

We appreciate the reviewers comment about this important point. While resting-state activity is widely used, the exact neural mechanism of it origin remains unresolved. The computational model we used in this study is unique in that it consists of individual neurons that can be tracked to estimate neuronal activity which underlies the generated BOLD signal and resting-state activity. It should be noted that the BOLD was estimated from the total synaptic input, so there was a strong correlation between BOLD and synaptic input in a given region. In addition, we also observed correlation between the extracellular K+, Na+/K+ pump and the estimated BOLD activity. The Na+/K+ pump activity reflect the metabolic activity of a given region (oxygen consumption) and this correlation further provide evidence that the BOLD activity measured in the model could capture the hemodynamics which is responsible for the BOLD activity in fMRI. To demonstrate the relation more clearly we created new figure which compares two essential variables of the model – extracellular potassium levels (the key driver of the infra-slow fluctuations, Krishnan et al., 2018) and synaptic currents as the primary source of BOLD (Logothetis et al., 2001, Viswanathan and Freeman 2007).

The figure demonstrates the similarity of all three measures in the dynamics, thereby creating similar correlation structure visible in functional connectivity. It has to be noted that the relation between BOLD and neural activity is very complex topic of its own and the identifying all contributing factors remain contested area (e.g. Gagnon et al., 2015, Lu et al., 2019). Indeed, it is one of our future plans to use biophysical modelling insights to contribute to this debate, but that needs different experimental data and is beyond scope of this manuscript.

We added the following to the description of our model in the section 4.1:

“We use the synaptic currents as the primary source of BOLD (Logothetis et al., 2001, Viswanathan and Freeman 2007). In particular, BOLD signal was created for each cluster first by averaging total synaptic input for all excitatory neurons in 100 ms windows and then it was convolved with a hemodynamic response function imported from Statistical Parametric Mapping (SPM) package (Ashburner et al., 2014). In addition, we also observed correlation between the extracellular K+, Na+/K+ pump and the estimated BOLD activity (SI Fig. 15). The Na+/K+ pump activity reflect the metabolic activity of a given region (oxygen consumption) and this correlation further provide evidence that the BOLD activity measured in the model could capture the hemodynamics which is responsible for the BOLD activity in fMRI.”

We changed the following description of fig. 5 (formerly fig.4) in section 2.2:

“B2. FC of the model.“ changed to

“B2. BOLD FC of the model. The essential biophysical variables underlying the BOLD signal and their FC are visible in SI Fig. 15.”

We added the following figure in the supplementary information (in section 5.8)

Selected biophysical variables underlying the BOLD signal and their FC.

Figure 15: Biophysical variables underlying the BOLD. Panels A: extracellular potassium levels (essential for slow fluctuations, see Krishnan et al., 2018), Panels B: synaptic input (primary source of BOLD). Panels C: resulting BOLD (panels C) dynamics and functional connectivity in the computational model of human connectome. Left panels (1): signal envelope for each region (computed as absolute value of analytical signal obtained from Hilbert transform after the BOLD was filtered between 0.01-0.03 Hz). Middle panels (2) – functional connectivity of raw signal. Right panels (3) – spectral amplitude of signal averaged across all regions ( ± SD in gray). D/E/F: Cross correlation between K+/Synaptic input/BOLD.
